# Structure modeling hints at a granular organization of the Golgi ribbon

**DOI:** 10.1186/s12915-022-01305-3

**Published:** 2022-05-13

**Authors:** Karen M. Page, Jessica J. McCormack, Mafalda Lopes-da-Silva, Francesca Patella, Kimberly Harrison-Lavoie, Jemima J. Burden, Ying-Yi Bernadette Quah, Dominic Scaglioni, Francesco Ferraro, Daniel F. Cutler

**Affiliations:** 1 Department of Mathematics, University College London, Gower Street, London, WC1E 6BT UK; 2 MRC Laboratory for Molecular cell Biology, University College London, Gower Street, London, WC1E 6BT UK; 3 Current address: iNOVA4Health, CEDOC-Chronic Diseases Research Center, NOVA Medical School, Universidade Nova de Lisboa, 1169-056 Lisboa, Portugal; 4 Current address: Kinomica, Alderley Park, Alderley Edge, Macclesfield, SK10 4TG UK; 5 Department of Biology and Evolution of Marine Organisms, BEOM, Stazione Zoologica Anton Dohrn, Villa Comunale, 80121 Naples, Italy

**Keywords:** Golgi apparatus, Golgi ribbon, Organelle structure, Weibel-Palade body, Mathematical modeling

## Abstract

**Background:**

In vertebrate cells, the Golgi functional subunits, mini-stacks, are linked into a tri-dimensional network. How this “ribbon” architecture relates to Golgi functions remains unclear. Are all connections between mini-stacks equal? Is the local structure of the ribbon of functional importance? These are difficult questions to address, without a quantifiable readout of the output of ribbon-embedded mini-stacks. Endothelial cells produce secretory granules, the Weibel-Palade bodies (WPB), whose von Willebrand Factor (VWF) cargo is central to hemostasis. The Golgi apparatus controls WPB size at both mini-stack and ribbon levels. Mini-stack dimensions delimit the size of VWF "boluses” whilst the ribbon architecture allows their linear co-packaging, thereby generating WPBs of different lengths. This Golgi/WPB size relationship suits mathematical analysis.

**Results:**

WPB lengths were quantized as multiples of the bolus size and mathematical modeling simulated the effects of different Golgi ribbon organizations on WPB size, to be compared with the ground truth of experimental data. An initial simple model, with the Golgi as a single long ribbon composed of linearly interlinked mini-stacks, was refined to a collection of mini-ribbons and then to a mixture of mini-stack dimers plus long ribbon segments. Complementing these models with cell culture experiments led to novel findings. Firstly, one-bolus sized WPBs are secreted faster than larger secretory granules. Secondly, microtubule depolymerization unlinks the Golgi into equal proportions of mini-stack monomers and dimers. Kinetics of binding/unbinding of mini-stack monomers underpinning the presence of stable dimers was then simulated. Assuming that stable mini-stack dimers and monomers persist within the ribbon resulted in a final model that predicts a “breathing” arrangement of the Golgi, where monomer and dimer mini-stacks within longer structures undergo continuous linking/unlinking, consistent with experimentally observed WPB size distributions.

**Conclusions:**

Hypothetical Golgi organizations were validated against a quantifiable secretory output. The best-fitting Golgi model, accounting for stable mini-stack dimers, is consistent with a highly dynamic ribbon structure, capable of rapid rearrangement. Our modeling exercise therefore predicts that at the fine-grained level the Golgi ribbon is more complex than generally thought. Future experiments will confirm whether such a ribbon organization is endothelial-specific or a general feature of vertebrate cells.

**Supplementary Information:**

The online version contains supplementary material available at 10.1186/s12915-022-01305-3.

## Background

**Fig. 1:**
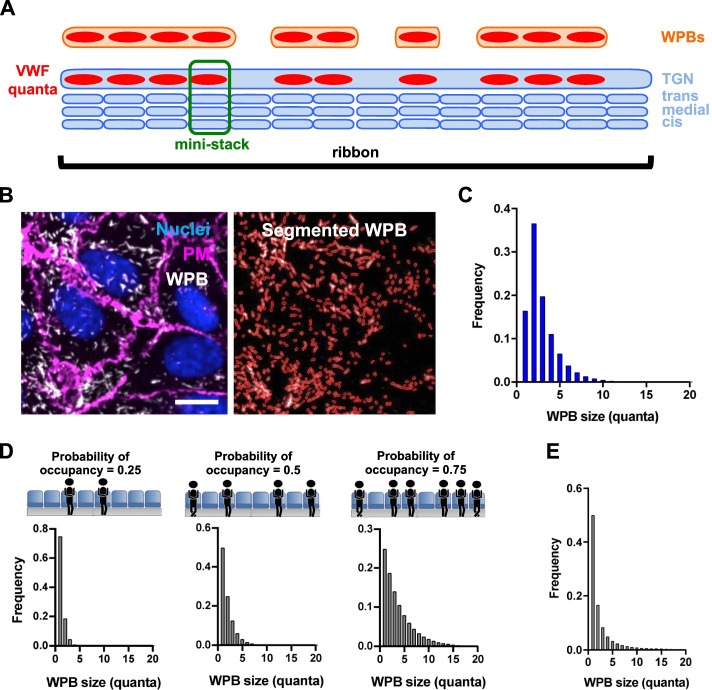
Modeling the Golgi ribbon as a linear array of mini-stacks. **A** Schematic representation of WPB biogenesis at the Golgi. In the context of the Golgi ribbon, the size of mini-stacks (highlighted in the green box) delimits that of transiting VWF quanta (red); at the Golgi exit site, the continuous lumen of the TGN allows co-packaging of adjacent quanta into nascent WPBs (orange). **B** On the left, a micrograph showing HUVECs immuno-stained for the plasma membrane (PM, with an anti-VE-cadherin antibody) and WPBs (with antibody which visualizes processed VWF at the Golgi and in WPBs) and counterstained for DNA (Nuclei) with Hoechst. On the right, WPBs have been segmented for quantification. Scale bar: 20 μm. **C** Measured WPB sizes (Additional file 1, Fig. S1A) were quantized by dividing the length of each organelle by the median quantum size (see text for details) and rounding to the nearest integer. **D** WPB size distributions as calculated from equation 1 at the indicated values of *p*, the probability of mini-stack occupancy by VWF quanta. **E** WPB size distribution calculated from Eq 1a, where *p* follows a probability distribution to account cell-to-cell variability in VWF expression

In multicellular organisms, such as plants and most animals, the Golgi apparatus is a multi-copy organelle with subunits, the mini-stacks, scattered throughout the cell, often in close contact with endoplasmic reticulum exit sites [1–3]. In contrast, in vertebrates mini-stacks link to each other into a tri-dimensional network, the Golgi ribbon [4–6]. This Golgi arrangement is dynamic, undergoing regulated disassembly/reassembly during mitosis, directed migration and polarized secretion [6]. The ribbon also disassembles in response to membrane depolarization, raised intracellular calcium, viral infection, autophagy induction and genotoxic insults [7–11]. The importance of the Golgi ribbon in the physiology of vertebrate cells is further underscored by accumulating observations showing that a host of human diseases, especially neurodegenerative, exhibit disruption of ribbon morphology, collectively referred to as “Golgi fragmentation” [12–14]. Nevertheless, the biological functions of the Golgi ribbon remain unclear [15], likely for two main reasons. First, understanding of the contributions of individual components of the complex molecular machinery involved in ribbon formation and maintenance is incomplete [16–28]. Second, a lack of cellular systems where the detailed structure of the ribbon is intimately linked to a quantifiable biological function has prevented much experimental probing of the effects of this Golgi organization on a measurable biological output. Recently, this latter situation changed. Weibel-Palade bodies (WPBs) are endothelial-specific uniquely rod-like secretory granules with lengths varying over a 10-fold range (0.5-5μm). WPB length reflects the dual-level structural arrangement of the vertebrate Golgi apparatus. The major WPB cargo, the pro-hemostatic secretory protein Von Willebrand Factor (VWF), is molded into boluses during its *cis-trans* passage through the functional subunits of the Golgi apparatus, the mini-stacks. Given the narrow size range of the mini-stacks, these VWF boluses also occur within a similar small range [29]. At the Golgi exit site, the continuous structure of the endothelial *trans*-Golgi network (TGN) allows co-packaging of adjacent VWF boluses into a linear array that drives the formation of nascent WPBs in an AP1 complex-dependent process [29–31]. The ribbon architecture of the Golgi apparatus thus determines the size of WPBs, which reflects the number of boluses co-packaged at biogenesis (Fig. 1A). For this reason, these VWF boluses were dubbed “quanta” [29].

WPB size determination can be summarized thus:

i) Mini-stack dimensions determine the size of VWF quanta. Changing the size of the mini-stacks also changes VWF quantum size and, as a consequence, WPB length.
ii) At the TGN, which in endothelial cells forms a continuous compartment spanning multiple mini-stacks, the ribbon configuration allows for VWF quanta occupying adjacent mini-stacks to be co-packaged into the same secretory granule. Unlinking of the ribbon, which also affects the TGN, results in WPB size distributions shifting to shorter lengths.
iii) The level of VWF expression controls the fraction of mini-stacks occupied by quanta; i.e., the number of VWF quanta formed. As a consequence, reducing VWF expression lowers the number of quanta within a Golgi ribbon, though not their size, resulting in fewer and shorter WPBs.

Statements *i-iii* are based on experimental evidence [29, 32].

This model of secretory granule biogenesis therefore invokes a key role for Golgi architecture, against which the level of VWF cargo controls both number and size of WPBs. Based on these premises, it is possible to mathematically model the effects of different organizations of the Golgi ribbon on WPB size and compare such simulations with experimental data obtained using a morphometric analytical workflow we developed to count numbers and measure the size of WPBs [29]. The aim of the present work was therefore to exploit the quantifiable readout of WPB size distribution to provide insight into possible mini-stack arrangements within the Golgi ribbon, which future experimental work might validate or disprove. The best-fitting model obtained with this approach indicates that the WPB size distribution observed in endothelial cells could be generated by a Golgi organization in which the ribbon physically integrates mini-stack monomers and dimers. Therefore, mathematical modeling of a system where the relationship between structure and function of the Golgi ribbon is clear and quantifiable raises the intriguing possibility that vertebrate mini-stack subunits are structurally - and presumably functionally - different.

## Results

The experimental data against which models were tested were generated with automated high-throughput morphometric analyses of cultured primary human umbilical vein endothelial cells (HUVECs). Immuno-stained WPBs were segmented to extract their length (Fig. 1B). Our model of WPB biogenesis (statements *i-iii*, above) is based on the central role of the mini-stack size in constraining the size of VWF quanta. We have shown that not only mini-stack and quantum size are similar but, importantly, changing the dimensions of mini-stacks impacts both VWF quantum and WPB sizes [29]. In control conditions, the median diameter of a Golgi mini-stack in HUVECs as measured in electron micrographs is 0.612 μm, which correlates with the median length of a VWF quantum (0.576 μm), determined by super-resolution microscopy [29]. In order to simplify mathematical modeling, we equated mini-stack length (*I*) to the median size of a VWF quantum and expressed WPB lengths in multiples of *l* (by rounding up to the nearest integer), thus transforming a distribution of WPB size from length (Additional file 1: Fig. S1A) to number of quanta (Q), or size class (i.e., 1Q-WPB, 2Q-WPB, etc.; Fig. 1C).

### Modeling the Golgi ribbon

To model how the Golgi generates this observed WPB size distribution, we started with the simplest assumptions (A):

- **A1**: the Golgi apparatus is a single, very long (effectively infinite) ribbon composed of linearly interlinked, identical mini-stacks, each of fixed length *l*^1^.
- **A2**: individual mini-stacks are occupied at random with a probability *p* by a VWF quantum.
- **A3**: VWF quanta occupying adjacent mini-stacks will be packaged together into the same WPB. If, for example, five contiguous mini-stacks are occupied, with empty mini-stacks at both sides, a WPB of length five quanta (a 5Q-WPB) will form at the TGN.

### The Golgi ribbon as a mini-stack linear array

Given the assumptions above, we can then model the ribbon as a “mini-stack linear array”, by denoting with *N* the number of quanta in a WPB (i.e., its size) and with *p* the probability of VWF quanta occupancy of mini-stacks across the Golgi ribbon, allowing calculation of the expected distributions of WPB lengths when *p* varies (Fig. 1D) with the following equation.

1 $$P\left(N=n\right)={p}^{n-1}\left(1-p\right),n=1,2\dots$$

Independently of the value of *p*, all WPB length distributions predicted with this model display a mode at *N* = 1 (Fig. 1D) and therefore do not simulate the experimental data that show a mode at *N* = 2 (Fig. 1C). Furthermore, the 2Q-WPB frequency in the experimental distribution, 0.366, is much higher than expected by the modeling at any value of *p*; in fact, the greatest proportion of 2Q-WPBs predicted by the model is 0.25 for *p* = 0.5 (Fig. 1D**,** middle panel).

### Variable mini-stack occupancy

We next considered whether the level of mini-stack occupancy might affect the relative frequency of the WPB size classes. Assuming that the rates at which WPBs are formed and that the rates at which they disappear from the cells, either because of exocytosis or degradation, are size-independent, one would expect cells with mini-stack occupancies close to 0.5 to have the most WPBs, since at very low occupancy there should be fewer WPBs, most of which should be 1Q-WPB, whereas at very high occupancy there should also be few WPBs but they should be close to the length of the Golgi ribbon.

Since cell-to-cell variability in the levels of VWF synthesis is observed in endothelial cells [33] and the HUVECs used in our lab are pooled from multiple donors, the probability of occupancy of Golgi mini-stacks by VWF quanta varies from cell to cell (Fig. 1B and Additional file 1: Fig. S1B). Trying to explain the observed WPB size distribution, captured from a cell population, by assuming a single value of *p* may therefore be an oversimplification. We thus modified the “the mini-stack linear array” predictive model by posing that *p* follows a probability distribution. If *p* is uniformly distributed between 0 and 1 within a population of endothelial cells, we obtain:

1a $$P\left(N=n\right)=\frac{1}{n\left(n+1\right)},n=1,2\dots$$

For every value of *p* this model predicts that the proportion of 2Q-WPBs is always ≤ 0.25 and the proportion of WPBs of length *N* strictly decreases with *N*. Therefore, even accounting for VWF expression variability does not explain the high proportion of measured 2Q-WPBs (Fig. 1E).

#### Partial conclusion 1

Our initial model is based on assumptions A1, A2 and A3. Assumptions A2 and A3 derive from our model of WPB size determination (statements *iii* and *ii*, respectively), which are experimentally supported [29]. Assumption A1**,** a very long, single ribbon formed by a “linear array of mini-stacks”, is therefore likely to be incorrect, failing to generate the observed excess of 2Q-WPBs when modeled (Fig. 1D and E).

### The Golgi as a collection of mini-ribbons

Alternatively, the ribbon might be formed from a collection of shorter, independent Golgi segments, mini-stack pairs included, which could act as preferential sites of formation for 2Q-WPBs. If *p* is sufficiently large, more 2Q-WPBs than 1Q-WPBs will form from paired mini-stacks; whereas at the lowest values of *p* only 1Q-WPBs will form. We tested whether a ribbon arrangement formed of short segments, a “mini-ribbon collection” model, might explain the observed frequency of 2Q-WPBs.

**Fig. 2:**
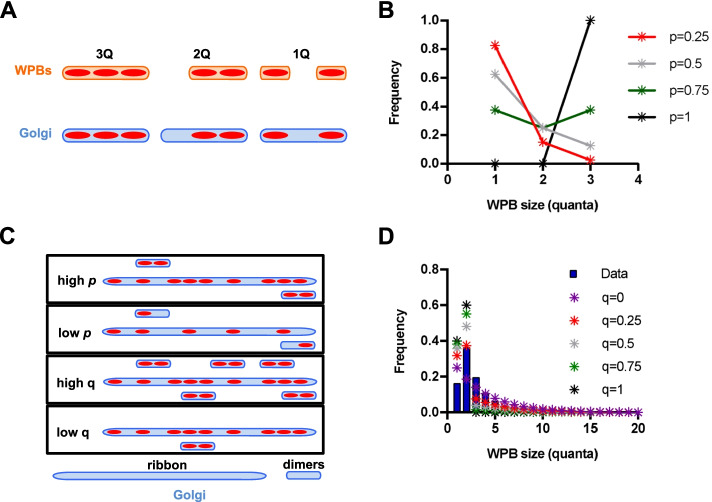
“Mini-ribbon collection” and “free mini-stack dimers plus linear ribbon” Golgi models. **A** Cartoon illustrating a model whereby the Golgi is composed of short ribbons of 3 mini-stacks (*l* = 3). WPBs of size 1Q, 2Q or 3Q can be made depending on probability of mini-stack occupancy, *p*. **B** For a Golgi made as described in **A**, the expected frequency distributions of 1Q-, 2Q- and 3Q-WPBs at the indicated values of *p* are shown. **C** Cartoon illustrating a model whereby the Golgi structure is made of free mini-stack dimers, present in proportion *q*, and a long linear ribbon. **D** based on the model in **C**, WPB size distributions calculated for *p* = 0.75 and the indicated values of *q* are shown (stars) in comparison to the measured size distribution (blue bars)

We modeled a Golgi made of a collection of mini-ribbons formed by elements of *l* = 3 (shown schematically in Fig. 2A) and *l* = 4, which give rise to WPBs of size 1Q-3Q and 1Q-4Q, respectively, with the frequencies shown in Table 1**.**

**Table 1:** Modeling of WPB proportions generated by Golgi mini-ribbons made by 3 or 4 mini-stacks. Numerical solutions for indicated values of *p* are shown in Fig. 2B and Additional file 2: Fig. S2A

	Relative proportion of WPBs of length 1	Relative proportion of WPBs of length 2	Relative proportion of WPBs of length 3	Relative proportion of WPBs of length 4
Golgi length *l* = 3	3*p*(1 − *p*)^2^ + 2*p*^2^(1 − *p*)	2*p*^2^(1 − *p*)	*p*^3^	0
Golgi length *l* = 4	4(1 − *p*)^3^*p* + 6*p*^2^(1 − *p*)^2 ^+2*p*^3^(1 − *p*)	3*p*^2^(1 − *p*)^2 ^+2*p*^3^(1 − *p*)	2*p*^3^(1 − *p*)	*p*^4^

In the first case, the maximum proportion of 2Q-WPBs predicted is 0.268 (for *p* = 0.75, Fig. 2B), while in the second it is always < 0.3 (Additional file 2: Fig. S2A).

#### Partial conclusion 2

A Golgi "ribbon" arranged in segments of linked but independent mini-stack trimers and tetramers does not explain the observed 2Q-WPB frequency. Moreover, such a “mini-ribbon collection” model predicts once again that the frequency of 1Q-WPBs should exceed that of 2Q-WPBs at any value of *p*, except 1 (Fig. 2B and Additional file 2: Fig. S2A), and thus do not correctly simulate the experimental data.

From these simple modeling exercises it appears that the only way to get a frequency of 2Q-WPBs exceeding 0.3 is to have a significant number of functionally paired mini-stacks. Ultimately, if *p* = 1, then having a Golgi ribbon composed of mini-stacks linked into functional units whose length distribution is equal to the WPB length distribution will obviously match the data. However, the above-mentioned cell-to-cell variability in VWF expression implies that at the cell population level the probability of mini-stack occupancy, *p*, cannot be 1.

### The “free mini-stack dimers plus linear ribbon” model

Let us then assume that the Golgi ribbon consists of a mixture of mini-stacks arranged into functional dimers plus long ribbon segments (depicted in Additional file 2: Fig. S2B and Fig. 2C). Equation 2 describes this “free mini-stack dimers plus ribbon” model, where *q* represents the proportion of mini-stacks existing as dimers.

2 $$P\left(N=n\right)=\left\{\begin{array}{c}\frac{\left(1-p\right)\left[1-p\left(1-q\right)\right]}{\left(1-p\right)+\frac{qp}{2}},n=1\\ {}\frac{p\left[{\left(1-p\right)}^2\left(1-q\right)+\frac{q}{2}\right]}{\left(1-p\right)+ qp/2},n=2\\ {}\frac{\left(1-q\right){p}^{n-1}{\left(1-p\right)}^2}{\left(1-p\right)+ qp/2},n=3,4\dots \end{array}\right.$$

In conditions where occupancy probability, *p,* is > 0.5 and *q* is sufficiently high, this model does predict an excess of 2Q-WPBs compared to 1Q-WPBs (Fig. 2D and Additional file 2: Fig. S2C). However, the “free mini-stack dimers plus linear ribbon” model predicts a proportion of 1Q- and 3Q-WPBs higher and lower, respectively, than that experimentally measured (Fig. 1C).

#### Partial conclusion 3

While the “free mini-stack dimers plus linear ribbon” model of Golgi organization might account for the high proportion of 2Q-WPBs in the experimental data, it does not account for the proportion of 1Q- and 3Q-WPB.

### 1Q-WPB instability

A major assumption built into our modeling so far is that WPBs of different sizes behave similarly; meaning that they are generated, stored and disappear from cells with identical kinetics. However, the unexpectedly low frequency of 1Q-WPBs might be due to their being less stable than larger WPBs. We modeled this possibility by assuming that for every currently forming WPB of length greater than one, there are *β* times as many WPBs of that length in the cell, whereas for 1Q-WPBs, there are *β’* times as many. We can therefore derive a value, *α*:

$$\alpha =\frac{\beta^{\prime }}{\beta }<1$$

where *α* describes the relative instability of 1Q-WPBs.

In these conditions, WPB frequency is given by:

3 $$P\left(N=n\right)=\left\{\begin{array}{c}\frac{\alpha \left(1-p\right)}{\alpha \left(1-p\right)+p},n=1\\ {}\frac{p^{n-1}\left(1-p\right)}{\alpha \left(1-p\right)+p},n\ge 2\end{array}\right.$$

We can fit the value of *p* for this distribution to the experimental data by minimizing the Kolmogorov-Smirnoff (KS) distance, that is the difference between the theoretical and experimental distributions for *n* ≥ 2. We find a best-fit value of *p* of 0.5692.

Using Eq. 3 for *n* = 1, we can then fix *α* to give us the correct proportion of 1Q-WPBs:

$$\alpha =0.5692\times \frac{0.1650}{\left[\left(1-0.5692\right)\times \left(1-0.1650\right)\right]}=0.2610.$$

**Fig. 3:**
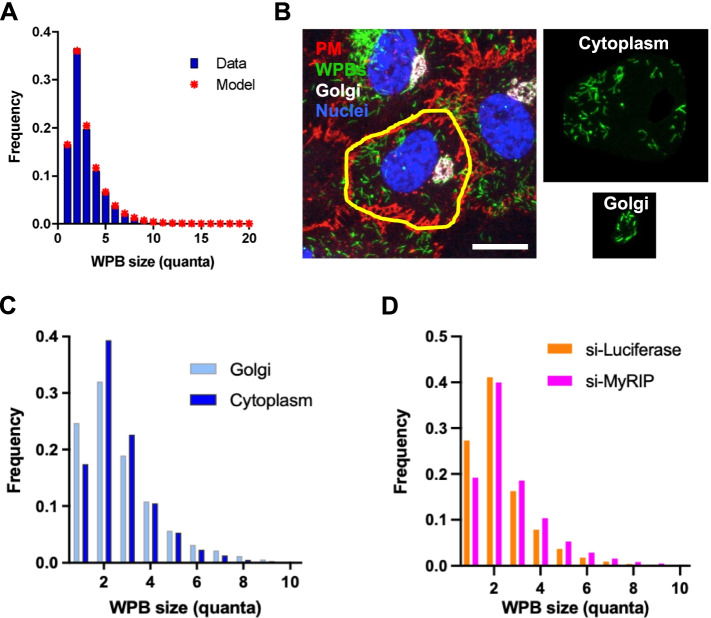
Instability of 1Q-WPBs. **A** Predicted WPB size distribution (stars) compared to real data (blue bars), where 1Q-WPBs display instability, *α*, greater than longer organelles. **B** HUVECs were fixed and processed to label the Golgi apparatus with an anti-TGN46 antibody and WPBs and cell boundaries as described in Fig. 1B. The Golgi area was masked to identify newly-made WPBs and separate them from the cytoplasmic organelles (panels on the right); scale bar: 20 μm. **C** WPB size distributions at the Golgi and in cytoplasm measured after the image segmentation illustrated in **B**. **D** WPB size distribution in control (si-Luciferase) and MyRIP-depleted (si-MyRIP) cells

For these values of *p* and *α*, the model-predicted and the experimental distributions do indeed show excellent agreement (Fig. 3A).

These results prompted us to further examine the possibility of 1Q-WPBs’ instability. If 1Q-WPBs are preferentially lost after biogenesis, they might be present in higher proportions where they are assembled; i.e. near and at the Golgi. We imaged and separately quantified the size of WPBs present in the vicinity of the Golgi versus in the rest of the cytoplasm. The peri-Golgi pool of WPBs indeed shows a higher fraction of 1Q-WPBs compared to those imaged in the cell periphery (Fig. 3B and C). These data raise the question of potential mechanisms underlying the differential depletion of the 1Q-WPBs. Endothelial cells control which sizes of WPBs are selected for exocytosis [34]. Therefore, one possibility is that 1Q-WPBs disappear by exocytosis. Release of VWF stored in WPBs occurs either acutely following exposure to agonist (stimulated exocytosis) or tonically, in the absence of any stimulus (basal exocytosis) [35]. WPBs are anchored to actin fibers adjacent to the cell membrane via the effectors Rab27 and MyRIP. Ablation of either of these proteins increases the release of VWF through basal exocytosis [36]. Does basal release differentially affect WPBs of different size classes? Depleting cells of MyRIP increases the fraction of long WPBs (Additional file 3: Fig. S3A and S3B), changing WPB size distribution (Fig. 3D). The reduced proportions of 1Q-WPBs indicate that secretory granules of this size class are preferentially depleted by basal exocytosis, and thus more readily disappear from the cell than longer organelles. The relative peri-Golgi enrichment of 1Q-WPBs and their differential propensity to undergo basal exocytosis thus may, at least partly, explain the observed secretory granule size distribution.

#### Partial conclusion 4

1Q-WPBs are more prone to basal exocytosis than longer WPBs.

### Stable mini-stack dimers

**Fig. 4:**
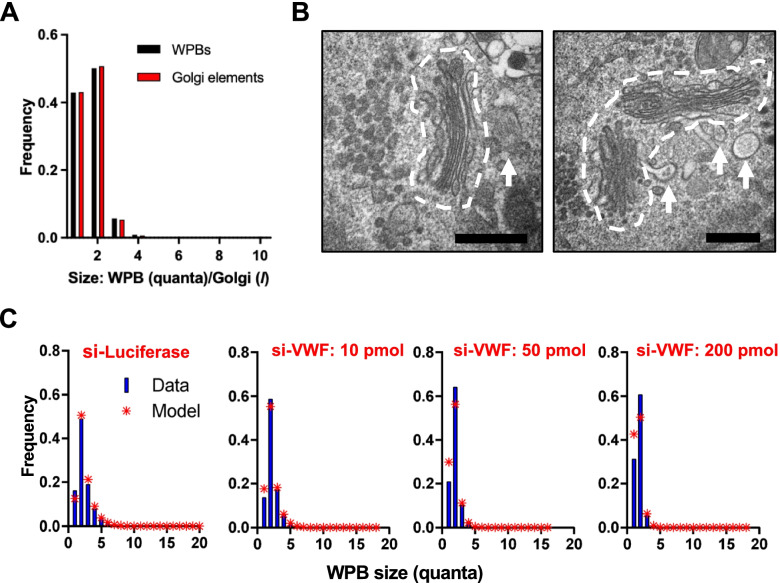
Stable mini-stack dimers**. A** After secretory granule depletion by PMA, cells were allowed to replete their WPB pool in nocodazole. Size distributions of Golgi elements and WPBs were measured. **B** Electron micrographs of HUVECs treated with nocodazole for 24 h. Dispersed Golgi mini-stacks (dashed outline) were observed as monomers (left) or dimers (right); arrows point at forming WPBs; scale bars: 500 nm. **C** Predicted length distribution of WPBs (red stars), maintaining *α* constant between datasets, compared to real data (blue bars) in control (si-Luciferase) and different levels of VWF expressing cells (si-VWF); note: the higher the amount of siRNA targeting VWF, the lower its expression

Our WPB biogenesis model posits that organelle length is determined by VWF quantum occupancy of adjacent mini-stacks (statement *iii*). The “free mini-stack dimers plus a long ribbon” model tested earlier, while failing to explain the frequencies of 1Q- and 3Q-WPBs, however suggested that a large proportion of mini-stack dimers within the ribbon might explain the measured frequency of 2Q-WPBs. We therefore explored whether we could detect the occurrence of mini-stack dimers in our system. Microtubules, their regulators and dynein motors are necessary for mini-stack tethering and Golgi ribbon formation [18, 22, 23]. Nocodazole depolymerizes microtubules, leading to ribbons unlinking into mini-stacks [22, 37]. We thus set out to quantify the effects of nocodazole treatment on WPB size distribution. Exposure to the strong secretagogue PMA (phorbol 12-myristate, 13-acetate) clears cells of pre-existing WPBs (Additional file 4: Fig. S4A) [38]. We implemented this treatment followed by recovery in the presence of nocodazole for 24h (Additional file 4: Fig. S4B). Compared to that of DMSO-pretreated/nocodazole-chased cells, the WPB size distribution of PMA-pretreated and nocodazole-chased cells shows a dramatic fall in the WPB classes > 2Q, compensated by a marked increase in 1Q-WPBs; remarkably, the proportion of 2Q-WPBs was unchanged (Additional file 4: Fig. S4C). Golgi fragments generated by nocodazole treatment are scattered throughout the cell (Additional file 4: Fig. S4B), facilitating their morphometric quantification. Golgi elements were thus measured and rounded up to the nearest multiple of *l*, as done for WPBs. This analysis revealed that Golgi elements were almost exclusively of sizes 1*l* and 2*l*, closely matching the distribution of WPB size classes (Fig. 4A). These data are consistent with repeated observations that the Golgi ribbon is necessary to the formation of long WPBs [29, 32, 39]; and, most importantly, they strongly suggest that even in the absence of microtubules, the Golgi of endothelial cells is not reduced exclusively to individual mini-stacks, but that around 50% of the Golgi objects generated by nocodazole treatment are of a size consistent with mini-stack dimers.

Inspection of the ultrastructure of the Golgi apparatus indeed showed the frequent occurrence of paired mini-stacks following nocodazole treatment, indicating they are stable structures in the absence of microtubules (Fig. 4B and Additional file 4, Fig. S4D).

#### Partial conclusion 5

In the absence of microtubules, the endothelial Golgi apparatus disassembles into monomer and stable dimer mini-stacks.

Previously, we have shown that reduction in VWF levels is reflected in the production of shorter and fewer WPBs (see statement *iii* regarding WPB size determination and reference [29]). Based on these observations, if mini-stack monomers and dimers were to persist in the ribbon, lowering VWF levels should increase the frequency of 1Q-WPBs and possibly affect the frequency of 2Q-WPBs less than that of longer organelles. We tested this expectation by treating HUVECs with different amounts of VWF-targeting siRNA to gradually reduce VWF cellular levels and therefore *p* (Fig. 4C). As expected, we find that 1Q-WPB frequency increases with the reduction of VWF levels and, strikingly, even when VWF levels are at their lowest (Fig. 4C, si-VWF: 200 pmol), the proportion of 2Q-WPBs remains high (blue bars), an indication that mini-stack dimers may be a preferred unit of WPB assembly. For each condition, we also modeled WPB frequencies (red stars) as done in Fig. 3A. We note that the model fits are not as good as in Fig. 3A, because we have required *α* to be constant across all four conditions (see Methods for details), where it thus has to explain more data. Allowing *α* to vary between the conditions results in much better fits^2^ but we could not justify this assumption biologically. These results are consistent with the possibility that the excess of 2Q-WPBs reflects the presence of functionally different mini-stack dimers within the ribbon, which would therefore be assembled from mini-stack monomers and these stable dimers. From this, it follows, again, that assumption A1 (mini-stacks are identical) is not likely to be true. VWF quantum co-assembly during WPB formation may therefore not be a purely random process, which would require modification of assumption A2 in its present formulation.

#### Partial conclusion 6

Reduction of cellular VWF does not impact 2Q-WPB frequency as much as that of longer organelles, indicating that stable dimers may persist in the assembled ribbon and provide a preferred site for WPB formation.

### The ribbon as a mixture of mini-stack monomers and dimers

Based on the findings above, we tried to assess how strong the preference for the mini-stack dimer vs monomer configurations might be by modeling binding rates of Golgi elements. We assumed that in the presence of intact microtubules, the Golgi elements continuously undergo a linking/unlinking process (“breathing”); that the rate at which two elements join is independent of their size; and that unlinking is slower for mini-stack dimers.

**Fig. 5:**
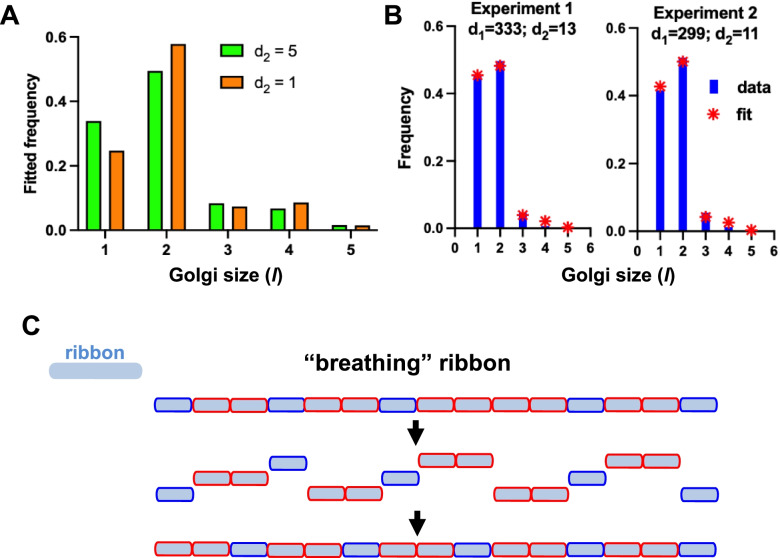
Mini-stacks organized into a “breathing” ribbon. **A** Modeling interactions among mini-stacks; binding and unbinding rates (reaction scheme in Additional file 6, A) were calculated by solving a system of differential equations (Additional file 6, B). Two examples of fitted frequencies of Golgi of size 1 - 5 *l* (i.e., 1 - 5 mini-stacks) obtained by posing r = 0.001, *d*_*1*_ = 100, total number of mini-stacks = 100000 and *d*_*2*_ at the indicated values. **B** Relative frequencies of Golgi pieces of different lengths (in mini-stacks) from two experiments with nocodazole-treated cells, together with the predictions from the model with the best fit values of *d*_*1*_ and *d*_*2*_ (each fitted to the nearest whole number). **C** A “breathing” Golgi ribbon model is schematized. Occurrence of weak bonds between monomers and stable dimers predicts their dynamic linking/unlinking and possible rearrangement within the ribbon. The model shows that the number of stable mini-stack dimers is the same as that of monomers as measured in our experiments (Fig. 4A)

Let the binding rate be *r*, and the unbinding rate *d*_*1*_ if it does not split a stable dimer, and *d*_*2*_ < *d*_*1*_ if it does. To simplify the modeling, we assume that two neighboring monomers always form a dimer, so that in longer sections of the ribbon, dimers can be separated, at most, by one monomer. We note that tetramers can have two forms either consisting of two coupled dimers or a dimer with a monomer on either side. To make the system of equations finite (see below), we also ignore portions of ribbon with lengths > five. We let the number of portions of length n be *l*_*n*_, for n = 1 to 5, and let M be the total number of mini-stacks. We denote by *l*_*4*_, the number of tetramers consisting of two dimers and *l*_*4*_*’* the number of tetramers consisting of a dimer and two monomers. There are two types of pentamers: each has two dimers and one monomer, but the monomer can be on the end or in the middle. These need to be considered separately, since they have different unbinding rates to the two types of tetramer (Additional file 5: Fig. S5). We denote the number of pentamers with a monomer on the end by *l*_*5*_ and the number with a monomer in the middle by *l’*_*5*_. We thus obtain a reaction system (Additional file 6: A). We can also represent the evolution of the mean numbers of each type of Golgi piece by differential equations, see Eq 4 (Additional file 6: B). The final values of the numbers of Golgi fragments of different lengths are given by the steady state values of these equations. Depending on the values of the parameters *r*, *d*_*1*_, *d*_*2*_ and the total number of mini-stacks, the distributions can peak at dimers and can have trimers either more or less frequent than tetramers. This is illustrated by stochastic simulations of the system of reactions, changing the value of the unbinding rate *d* (Matlab code provided in Additional file 7). Two examples are shown: in Fig. 5A, pieces of length three are more abundant than those of length four in one simulation, whereas in the other the opposite is true.

Whether tighter linking of dimers leads to an abundance of Golgi pieces of length four (and potentially all multiples of two) therefore depends on the parameters of the system. We now fit the data. We are interested in the steady state frequencies of Golgi pieces of different length; therefore, in the model, we are free to choose both the total number of Golgi pieces (although this should be large to accurately sample the predicted frequencies) and the rate at which the system reaches equilibrium. The only combinations of parameters that should affect the steady state frequencies are *r*M*/d*_*1*_ and *d*_*1*_*/d*_*2*_. We therefore only need to explore different values of these two dimensionless parameter combinations. We can do so by arbitrarily fixing M (subject to it being large enough to get good sampling) and *r* and varying *d*_*1*_ and *d*_*2*_. Therefore, we fix *r* = 0.001 and M = 100,000 (modeling a population of cells), both of these are dimensionless, and fit *d*_*1*_ and *d*_*2*_ to the data on proportions of Golgi of different lengths.

We show in Fig. 5B the distributions of lengths of Golgi fragments from two actual experiments, in which cells were treated with nocodazole, and from the model fit to those experiments. The reason we used these data is that in the control conditions, i.e. in the context of a linked ribbon, individual Golgi elements cannot be accurately measured, while this is possible after nocodazole treatment (Fig. 4A and Additional file 4: Fig. S4B). These simulations show a good fit to the experimental data. The calculated values of the dissociation rates based on the experimental data are *d*_*1*_ = 333 and 299 and *d*_*2*_ = 13 and 11 (chosen to minimize KS distance), respectively. Thus, the prevalence of dimers may be explained by a striking 30-fold difference in mini-stack dissociation rates. We note that the actual values of *d*_*1*_ and *d*_*2*_ depend on our choice of the dimensional time corresponding to a unit of dimensionless time, which we cannot estimate from the steady state proportions of Golgi lengths.

Having approximately 50% of the Golgi ribbon composed of (possibly 30-fold) more-tightly linked dimers might explain why nocodazole fails to unlink the Golgi entirely into monomers (Fig. 4A-B and schematized in Additional file 4: Fig. S4D). Unlike the “free mini-stack dimers plus ribbon” model (Fig. 2C-D and Additional file 2: Fig. S2B-S2C), where we considered mini-stack dimers as existing separate from the ribbon, by having 50% of Golgi objects as functional dimeric mini-stacks that are incorporated into the ribbon (Fig. 5C**)**, it is possible to assemble longer organelles on these.

#### Partial conclusion 7

Modeling mini-stack binding finds that considerably different rates of dissociation between mini-stacks might account for the observed dimer stability even in the absence of microtubules.

## Discussion

Weibel-Palade bodies may represent a unique cellular system in organelle size-control due to the two-tiered functioning of the Golgi apparatus in their biogenesis. Golgi mini-stack dimensions constrain the size of VWF quanta, while the Golgi ribbon architecture controls co-packaging of VWF quanta into nascent secretory granules [29]. As a consequence, the structural arrangement of a biosynthetic compartment, the Golgi, is closely reflected by the size of the organelles it generates, the WPBs. The size distribution of WPBs thus provides a proxy for the architecture of the Golgi apparatus. Taking advantage of this unique cellular system we generated a formal description of the structural organization of the Golgi apparatus. Hypotheses about the Golgi ribbon structure, translated into mathematical modeling, predicted WPB size distributions that were tested against those actually measured using an image analysis workflow that we previously employed in a series of studies [29, 32, 34, 39–42].

The measured size distribution of WPBs posed two challenges to modeling of the Golgi ribbon structure. First, why are 1Q-WPB depleted with respect to other size classes? Second, what kind of mini-stack arrangement can explain the high frequency of 2Q-WPBs with respect to other size classes?

For the Golgi ribbon structure to be consistent both with these data, plus what is known of WPB biogenesis, its model had to be refined from that of a single very long ribbon made by linearly linked mini-stacks, through to that of an ensemble of short ribbon stretches or a combination of mini-stack dimers and a ribbon, finally to an organization involving a mixture of mini-stack monomers and dimers.

For 1Q-WPBs, mathematical modeling suggested their low frequency might be explained by a selectively shorter residence time. Experimental testing of this hypothesis indeed indicates that 1Q-WPBs undergo basal exocytosis at higher rates than longer WPBs. In agreement with this finding, we had previously found that short WPBs are more prone to exocytosis [29, 32]. Of note, the modeling exercise deployed in this study allowed us to narrow down this effect to 1Q-WPBs, as those preferentially selected for basal secretion.

Regarding the second question, the observed steady-state frequency of 2Q-WPBs can, in principle, be explained by assuming that the Golgi ribbon contains a large proportion of mini-stack dimers. This prompted experimental testing combining treatment with PMA and nocodazole, which deplete the cellular pool of WPBs and break up the ribbon into mini-stacks, respectively. Strikingly, in these conditions we observed that 1Q- and 2Q-WPBs were almost exclusively made, with their size matched by that of Golgi elements. Since there is no experimental evidence from the literature for quantization of mini-stack size, we had to conclude that microtubule disruption generates Golgi elements existing as mini-stack monomers and dimers. This conclusion was confirmed by electron microscopy observations. Thus, a structural basis must exist to prevent mini-stack dimer dissociation. Based on the ratio of mini-stack monomers and dimers following nocodazole treatment, we ran simulations that resulted in an estimated “strength” of the link between mini-stacks in a dimer being approximately 30-fold greater than that of monomers with dimers in the context of a ribbon (Fig. 5 and Additional files 5, 6 and 7). The modeling of our experimental data thus fits with a picture of the endothelial Golgi ribbon as a dynamic structure formed by mini-stack monomers and very stable dimers, accounting for the measured frequency of 2Q-WPBs.

Intriguingly, actin-dependent paired mini-stacks were described in insect cells, displaying a Golgi organization of scattered elements, typical of invertebrate animals [43]. Following actin-depolymerizing drug treatment, Kondylis and co-workers found that the number of Golgi elements increased by 50%, as would be expected if, similar to nocodazole-treated HUVECs, only half of the mini-stacks were dimers (refer to Fig. 2B in reference [43]). Moreover, in HUVECs individual mini-stack incorporated into dimers tend to sit at an angle with respect to each other (Fig. 4B); a similar configuration to that observed in insect mini-stack pairs (see Fig. 5A-B in reference [43]). These authors further showed that in mammalian HeLa cells, pretreated with nocodazole to unlink the ribbon, inhibition of actin polymerization caused Golgi elements’ scission, suggesting the likely presence of mini-stack pairs also in mammalian cells [43]. One can speculate that mini-stack dimers may be a widespread feature of the Golgi apparatus of animal cells.

While our modeling exercise and the experimental manipulations thereby prompted support the presence of stable, microtubule-independent mini-stack dimers in mammalian endothelial cells, at this stage it is not clear how these dimers are kept together. Based on the above-mentioned work [43], the actin cytoskeleton is the likely driver of stable mini-stack dimer formation. This is consistent with the evidence that either actin depolymerization or hyper-polymerization at the Golgi results in ribbon fragmentation [24, 25]. However, precisely which of its regulators are involved remains to be experimentally established. Golgi membranes certainly provide a scaffold for many candidates [44].

Adopting WPB size distribution as a proxy for modeling the Golgi ribbon organization thus paints an unanticipated picture of this architecture. Our best-fitting model is consistent with a ribbon made from similar numbers of mini-stack monomers and dimers undergoing continuous linking/unlinking: a “breathing” ribbon model (Fig. 5C). This “breathing” would make the Golgi ribbon a highly dynamic structure, capable of promptly responding to signals that require its rearrangement, such as re-positioning and re-orientation during migration and directed secretion [23, 45, 46]. It is possible that such a structural organization may not be universal to vertebrate cells, but rather reflect an endothelial adaptation of the Golgi ribbon to the need for WPB production, providing a flexible system capable of modulating the size of these organelles in response to physiological and pharmacological cues [29, 34, 39, 41]. However, the above-mentioned indirect evidence of similar dimers being present in HeLa cells [43] suggests that this might be a widespread feature. In the “breathing” ribbon model (Fig. 5C), formation of a dimer prevents a similar strong interaction of either of the constituent mini-stacks with another monomer. This implies that the machinery responsible for stable dimer formation must segregate at the location of the mini-stacks' interaction (Additional file 5: Fig. S5**)**; that is, at a site on the rims of the cisternae. Polarization of molecular machinery within organelles has been described. For instance, MyRIP, which interacts with F-actin, localizes at one tip of WPBs [36]. In mini-stack monomers, Golgi structural proteins localize at the rims of the cisternae [47] and one possibility is that lipid-mediated phase separation and consequent protein spatial restriction [48] may segregate the machinery involved in dimer stabilization.

Our modeling-based prediction of functionally distinct and stable mini-stack dimers does not shed light on the physical nature of their embedding into the ribbon superstructure. Fluorescence recovery after photo-bleaching (FRAP) experiments firmly established that cisternal membranes of neighboring mini-stacks are continuous [16, 17, 49]. Therefore, a question raised by our “breathing” model of the Golgi ribbon is that of what molecular or physico-chemical characteristics locally define the mini-stack dimers, while allowing their membrane continuity with adjacent mini-stacks. Our findings may prompt further studies to investigate this issue.

### Limitations of the study

Being based on incompletely defined biological processes, the simplistic modeling exercises deployed in the present study may incompletely capture the biological complexity underlying the phenomena we formalized mathematically.

For example, the Golgi ribbon models here presented are deduced exclusively by the behavior of a single biological system, WPBs. This is due to the fact that, to our knowledge, this is the only system for which a relationship between the structure of the biosynthetic compartment (the Golgi ribbon) and size of secretory output (WPBs) is established [29, 32]. In addition, while our model of WPB biogenesis has withstood several experimental tests [29, 32, 39], it does not - for example - consider whether, given their size, limited lateral mobility of VWF quanta within the continuous lumen of the TGN may favor the observed high frequency of 2Q-WPBs. Actually, time-lapse imaging indicates that VWF aggregations within the Golgi are very mobile (Movie S1 in reference [29]). However, due to limitations in resolution it is not clear whether this VWF mobility is inherent to quanta or rather reflects the movement of VWF quantum-containing portions of the ribbon relative to each other. Future, more sophisticated imaging approaches may help decipher the nature of the VWF mobility observed at the Golgi and, if relevant, lead to a refined WPB biogenesis model. Finally, VWF residence time at the TGN, determined by the balance between its rates of arrival (anterograde traffic) at, and of departure from (WPB budding) the TGN, may impact quanta co-packaging. VWF kinetics at the Golgi is however poorly defined and therefore not considered in our WPB biogenesis model.

Our models of the Golgi ribbon may also be oversimplified. In endothelial cells, the TGN is present as a continuous compartment spanning adjacent mini-stacks within the ribbon [29]. This structural organization of the TGN, embedded in our WPB biogenesis model, is also assumed with respect the Golgi structures we hypothesize in the present study. However, it is not established whether TGN continuity is a general feature of all vertebrate cell types. Also, the intermediate compartment (IC) and the recycling endosome (RE) networks were recently proposed to participate in Golgi ribbon formation by providing stable templates for the organization of mini-stacks into the ribbon arrangement [50]; this model of ribbon organization was not considered in our work.

Finally, our modeling of binding rates may not be accurate since quantitation of mini-stack monomers and dimers was possible only in nocodazole treated cells. Within an assembled ribbon, the presence of microtubules and/or other molecular components may impact binding rates in ways we have not accounted for here.

## Conclusions

Our modeling exercise has provided insight into the dynamics of the smaller class of WPBs. Further detailed analysis - using the quantitative approach that we introduce in this study - of the roles of the known endothelial exocytic machinery would identify the molecular mechanisms regulating basal secretion and thus just how the 1Q-WPBs are preferentially released; a process potentially critical to setting the level of plasma VWF and thus thresholds for hemostasis, fundamental to both physiology and pathology [35].

Of more general interest and importance are the implications of this work regarding the Golgi apparatus and its architecture. Despite the mentioned limitations and the assumptions embedded in our mathematical models, these led us to the identification of stable, microtubule-independent, mini-stack dimers as a major component of Golgi apparatus. The “breathing” ribbon model raises obvious questions as to the molecular machinery involved in stable mini-stack dimer formation and mini-stack dynamics in the context of the ribbon. If this model does indeed reflect an actual structural arrangement, a systematic approach designed to identify the machinery controlling the architecture of the Golgi ribbon, taking advantage of WPB size distribution as proxy of the mini-stack linkage status, could further our understanding of how this remarkable structure is formed and, potentially, reveal how its fragmentation is linked to disease.

## Methods

### Cells

Human umbilical vein endothelial cells (HUVECs) pooled from donors of both sexes were obtained commercially from PromoCell or Lonza. Cells were expanded and used within 15 population doublings, corresponding to passage 3 to 4 after expansion by our lab and maintained in HUVEC Growth Medium (HGM): M199 (Gibco, Life Technologies), 20% Fetal Bovine Serum, (Labtech), 30 μg/mL endothelial cell growth supplement from bovine neural tissue and 10 U/mL Heparin (both from Sigma-Aldrich). Cells were cultured at 37 °C, 5% CO_2_, in humidified incubators.

### Reagents

#### Chemicals

All common reagents were from Merck (Sigma-Aldrich). Nocodazole (M1404), Phorbol 12-myristate 13-acetate (P8139) and DMSO (D2650) were from Merck (Sigma-Aldrich). Nocodazole and PMA were dissolved in DMSO to generate 10 and 0.1 mg/mL stock solutions, respectively. Antibodies used in this study were rabbit polyclonal antibody raised against the cleaved VWF pro-peptide region [51]; mouse monoclonal anti-VE-cadherin (BD Bioscience, Clone 55-7H1, cat. no. 555661); sheep anti-TGN46 (BioRad, cat. No, AHP500G). siRNAs targeting firefly Luciferase (5’-CGUACGCGGAAUACUUCGAdTdT-3’), human VWF (5’-GGGCUCGAGUGUACCAAAAdTdT-3’) and human MyRIP (5’-AAGGTGGGAATTATTATTTAA-3’ and 5’-CCAAATTTACTTCCCAATAAA-3’) were previously described and validated [36, 52, 53]. siRNAs were custom synthesized by Eurofins MWG Operon.

### Treatments

HUVECs were seeded on gelatin-coated NUNC 96-well plates (Thermo Fisher, cat.no. 167008) at 15000-20000 cells/well and maintained in HGM. Untreated cells were fixed after 48 h. In WPB repopulation experiments, 23 h after seeding, cells were fed with HGM supplemented with either DMSO or with PMA (100 ng/mL) for 1 h to elicit massive VWF secretion and deplete WPBs, followed by nocodazole incubation (1 μg/mL) for 24 h to allow WPB formation from separated Golgi mini-stacks. In the experiments involving VWF or MyRIP depletion, the indicated amounts of siRNAs were electroporated into one million HUVECs per reaction with an AMAXA Nucleofector II (Lonza), following the manufacturer’s instructions. Cells were then plated in 96-well plates as described above and cultured for 48 h. Cells were then rinsed twice with warm, fresh HGM and fixed by incubation with 4% formaldehyde in PBS (10 min, at room temperature).

### Immuno-staining and image acquisition

After permeabilization (with 0.2% TX-100 in PBS) and blocking (with 1 % bovine serum albumin in PBS), cells were incubated with primary antibodies, followed by secondary Alexa Fluor dye-conjugated antibodies (Life Technologies), both diluted in 1% BSA, 0.02% TX-100 in PBS. Nuclei were counterstained with Hoechst 33342 (Life Technologies, H3570). Imaging was carried out with an Opera High Content Screening System (Perkin Elmer), using a 40x air objective (NA 0.6). Nine fields of view per well in 8-16 replicate wells were acquired, corresponding to approximately 2400-4800 cells per condition. The size in μm of ~ 100000 WPBs was measured for each condition. For Golgi and cytoplasm WPB size determination, together with WPBs the Golgi apparatus and cell boundaries were visualized with anti-TGN46 and VE-cadherin antibodies. Imaging was carried out with a 63x (NA 1.3) oil immersion objectives on a Leica Microsystems TCS SP5 confocal system. Eleven stacks (0.5 μm z-step) were reduced to maximum intensity projections. Approximately, 6000 to 9000 WPBs were analyzed for each of three independent experiments.

### Image analysis

High-throughput morphometry (HTM), the analytical pipeline including image processing, organelle segmentation and measurement of morphological parameters, has been described in detail elsewhere [29]. Briefly, images of WPB (visualized with anti-VWF pro-peptide antibody) were segmented using Python (v2.7) (see script in Additional file 8). Data analysis was done using *R* (i386 3.1.0; script in Additional file 10). The size in μm of ~ 100000 WPBs was measured for each condition. WPB lengths were expressed as numbers of quanta as described in the Results section. For WPBs localized at the Golgi or in the cytoplasm, three experiments were carried out; WPB were segmented using a FIJI macro (Additional file 9) and their size analyzed with *R*, as described above.

### Electron microscopy

Nocodazole-treated cells were high-pressure frozen, free-substituted (HPF/FS) and processed as described previously [54]. Samples were imaged with FEI Tecnai 20 TEM using an Olympus-Soft Imaging System Morada camera.

### Sample size

For each condition the minimum number of cells imaged was from 72 fields of view (FOV), corresponding to 8 wells of 96-well plates. The number of independent biological replicates for each condition was 2 or 3, depending on the condition. To rule out experimenter’s bias, image acquisition (i.e., selection of FOV, etc.) and measurements taken from the images were fully automated. In pilot experiments (not reported in the present study), we sampled approximately 100000 WPB from control and treated cells and determined the statistical difference between these populations. We then took random samples of diminishing numbers of WPBs from these two groups and determined the point (10000 organelles) at which the significance of the difference fell below *P* ≤ 0.05. Using nocodazole to shorten the sizes of WPBs, and sampling as described above, the statistical power is 1 (the effect size of this treatment is approximately 2.02 measured by Cohen’s *d*). For the analyses reported in the present study, we therefore collected data from ~ 100000 WPBs for each condition.

### Mathematical modeling

To create the model fit for Fig. 3A, we used Eq. 3, for *n* ≥ 2, (see text). We initially ignored the frequency of 1Q-WPBs. The relative frequencies of WPBs of lengths 2Q, 3Q, 4Q… are then given by 1-*p*, *p*(1-*p*), *p*^2^(1-*p*), … For multiple values of *p* in the range [0,1], we generated this distribution and measured the Kolmogorov-Smirnoff distance (this is the maximal absolute difference between the cumulative distribution functions) to the experimental relative frequency distribution of WPBs of lengths 2Q, 3Q, 4Q… We found the value of *p* that minimized this distance. Then, using Eq. 3, for *n* = 1, (see text) with the optimal value of *p* already found, we fixed α to give us the exactly correct frequency of 1Q-WPBs.

To create the model distributions in Figs. 5A and B, we fixed values for *d*_*1*_ and *d*_*2*_ and then ran a code (Additional file 7) to numerically simulate the stochastic process described by the reactions given in Additional file 6: A. At a late time (t = 5000), we output the relative frequencies of the Golgi portions of length 1-5 mini-stacks. For Fig. 5B, we then computed the Kolmogorov-Smirnoff distance to the experimental values of those relative frequencies. We repeated the process for *d*_*1*_ = 1, 2, 3, …., 20 and *d*_*2*_ = 1, 2, …., 400 and found the values of *d*_*1*_ and *d*_*2*_ that minimized the distance. Note that had these values been at an extreme value of the range, we would have searched a broader set of values for *d*_*1*_ and/or *d*_*2*_.

To create the model fits for Fig. 4C, we fit values of *p* to the data from the control and three siRNA concentration experiments, as we did for Fig. 3A. We then used the frequencies of 1Q-WPBs to exactly determine α for each experiment. However, we assumed that α would not vary due to siRNA treatment, so that its values should be the same for all conditions. Thus, we took the weighted mean of the values of α that best fit each data set. We multiplied the value of α obtained for the luciferase siRNA condition by the total number of WPBs in that experiment and added the values of α obtained for VWF siRNA at 10-200 pmol, multiplied by the corresponding number of WPBs in each condition, then divided by the total number of WPBs over all four conditions. We then plotted the model predicted distributions with that single value of α and the values of *p* chosen for each experiment separately. Note that had we allowed α to vary between the conditions, the fits would have been considerably better.

## Supplementary Information

**Additional file 1: Fig. S1.** WPB size distribution measured from a population of endothelial cells. A WPB size distribution as measured by high-throughput morphometry (see Methods). B HUVECs show variability in VWF expression and WPB production. WPBs and nuclei were visualized as indicated in Fig. 1B; the Golgi was visualized with an anti-GM130 antibody; scale bar: 20 μm.

**Additional file 2: Fig. S2.** Further simulations based on the “mini-ribbon collection” and the “free mini-stack dimers plus ribbon” models. A Predictions of WPB size distributions at different probabilities of quantum mini-stack occupancy probability (*p*), where the Golgi is made exclusively of ribbons of length 4*l*. B A Golgi made of ribbons and mini-stack dimers, where the latter are present in proportion q; different q values are depicted. C Based on the model in B, expected WPB size distributions (stars) were calculated for the indicated mini-stack occupancy probability (*p*) and the proportion of the Golgi formed by dimers (q) and compared to the measured WPB size distribution (blue bars).

**Additional file 3: Fig. S3.** Shifts in WPB size distribution following increase in basal secretion. A Efficiency of MyRIP knockdown (300 pmol of each siRNA were used per reaction); means and ranges from two independent experiments are reported; Student’s t-test. B Morphometric analysis of WPB size shows that MyRIP knockdown increases the fraction of long organelles (defined as those > 2 μm); data shown are from one of the replicate experiments shown in A; statistical analysis was non-parametric (Mann-Whitney test).

**Additional file 4: Fig. S4.** Shifts in WPB size distribution following Golgi ribbon unlinking. A HUVECs were treated for 1 h with either DMSO or 100 ng/mL PMA and processed for immunofluorescence. PMA almost completely depletes the cellular pool of WPBs. Scale bar, 25 μm. B Representative micrograph of HUVECs pre-treated with PMA as in A to clear WPBs and then chased in nocodazole for 24 h; scale bar: 25 μm. C Size distribution of WPBs 24 h after nocodazole treatment, following pre-treatment with either DMSO (control) or PMA to deplete the organelles. The resulting populations of organelles following 24 h nocodazole treatment are newly-made WPBs plus those left after basal exocytosis, in the case DMSO; almost completely newly-made WPBs, in the case of PMA (see panel A). D Visualization of a hypothetical arrangement of the Golgi ribbon where stable mini-stacks dimers are independent of microtubules.

**Additional file 5: Fig. S5.** Modeling of mini-stack linking to form the Golgi ribbon. The possible mini-stacks associations up to pentamers are depicted (see text).

**Additional file 6.** A Reaction scheme for mini-stack linking and unlinking. B Equation system 4.

**Additional file 7.** Matlab code for numerical simulations of the reaction scheme described in Additional file 5 A.

**Additional file 8.** Python script for WPB segmentation.

**Additional file 9.** FIJI macro for Golgi and extra-Golgi WPB segmentation.

**Additional file 10: ***R* script for WPB size determination from images.

## Data Availability

The relevant data generated and analyzed in this study are included in this published article and its supplementary information. The original datasets used and analyzed for the present study are available from KMP and DFC on request.

## Abbreviations

WPB: Weibel-Palade Body
VWF: Von Willebrand Factor
TGN: *trans*-Golgi Network
ER: endoplasmic reticulum

## Footnotes

1 The infinite ribbon assumption is made to avoid cell-dependent variations; to reflect, like the WPB distribution, a cell population; and also to simplify probability calculations*.*

2 We note that the best fit value of *α* decreased with increasing siRNA concentration; data not shown.
