# Supplementary figures and images for: Structure modeling hints at a granular organization of the Golgi ribbon
Source: BMC Biol. 2022 May 13;20:111. doi: 10.1186/s12915-022-01305-3 (PMC9102599; doi:10.1186/s12915-022-01305-3)

**A**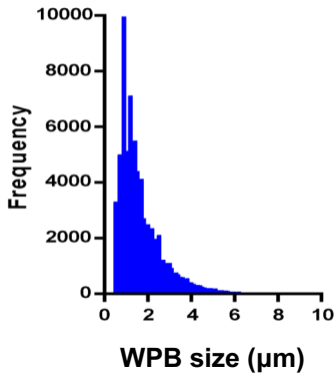**B**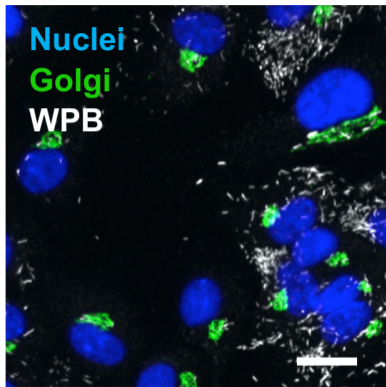

Supplement: Supplementary file 1 — Additional file 1: Fig. S1. WPB size distribution measured from a population of endothelial cells. A WPB size distribution as measured by high-throughput morphometry (see Methods). B HUVECs show variability in VWF expression and WPB production. WPBs and nuclei were visualized as indicated in Fig. 1B; the Golgi was visualized with an anti-GM130 antibody; scale bar: 20 μm. [file 12915_2022_1305_MOESM1_ESM.pdf]

**A**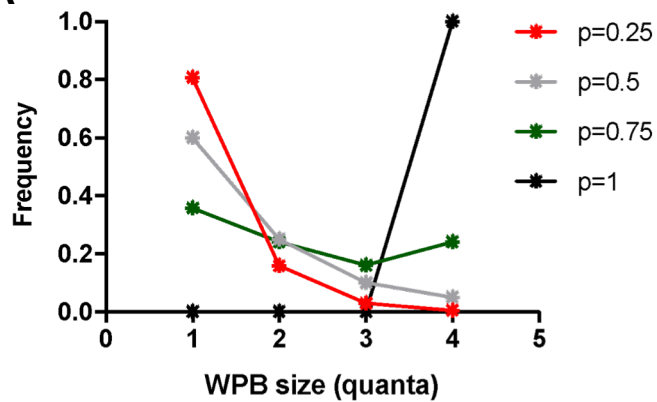**B**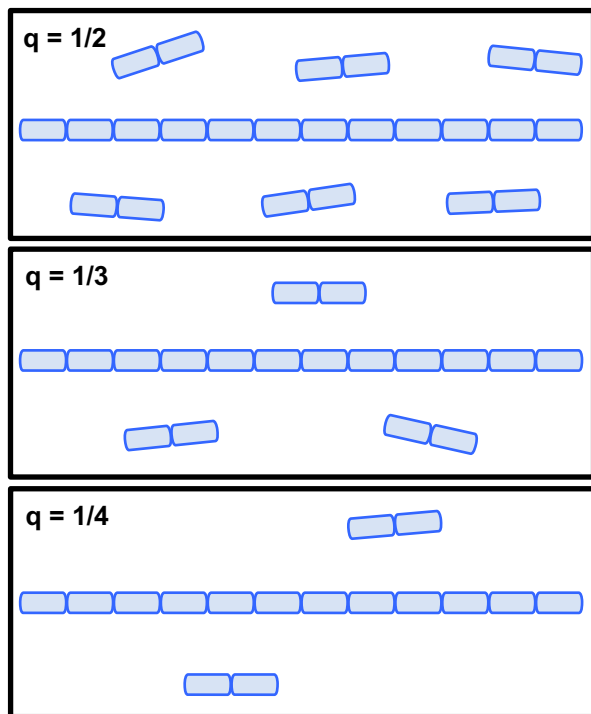**C**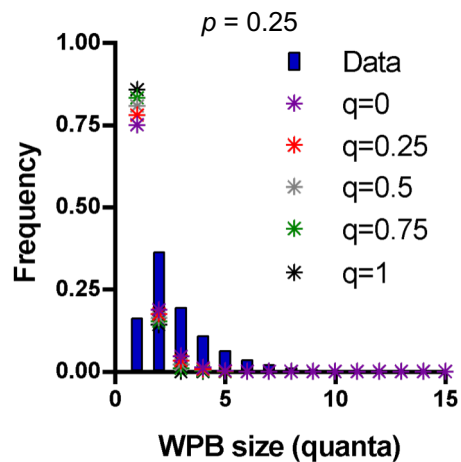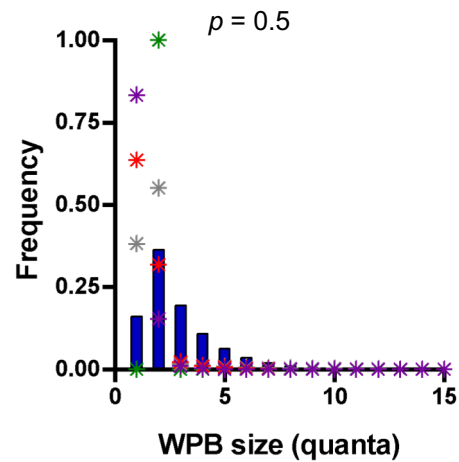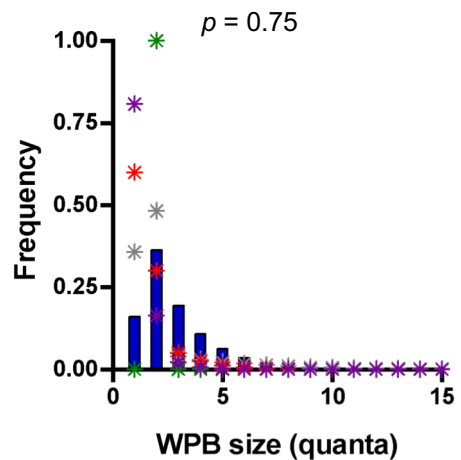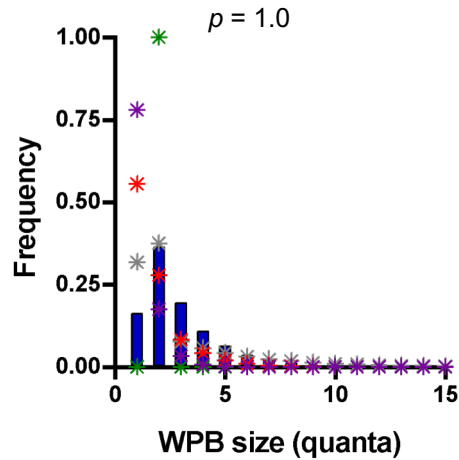

Supplement: Supplementary file 2 — Additional file 2: Fig. S2. Further simulations based on the “mini-ribbon collection” and the “free mini-stack dimers plus ribbon” models. A Predictions of WPB size distributions at different probabilities of quantum mini-stack occupancy probability (p), where the Golgi is made exclusively of ribbons of length 4l. B A Golgi made of ribbons and mini-stack dimers, where the latter are present in proportion q; different q values are depicted. C Based on the model in B, expected WPB size distributions (stars) were calculated for the indicated mini-stack occupancy probability (p) and the proportion of the Golgi formed by dimers (q) and compared to the measured WPB size distribution (blue bars). [file 12915_2022_1305_MOESM2_ESM.pdf]

**A**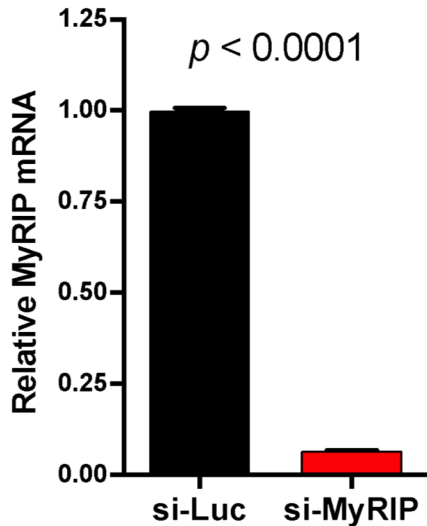**B**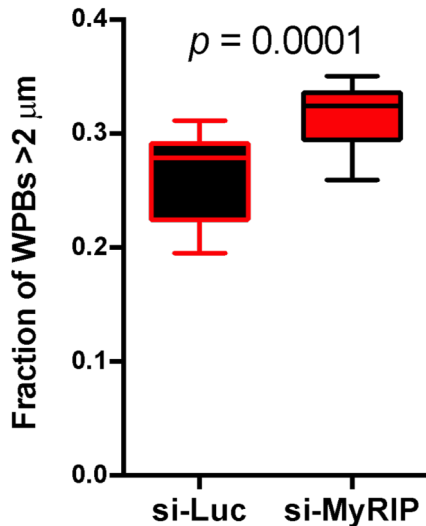

Supplement: Supplementary file 3 — Additional file 3: Fig. S3. Shifts in WPB size distribution following increase in basal secretion. A Efficiency of MyRIP knockdown (300 pmol of each siRNA were used per reaction); means and ranges from two independent experiments are reported; Student’s t-test. B Morphometric analysis of WPB size shows that MyRIP knockdown increases the fraction of long organelles (defined as those > 2 μm); data shown are from one of the replicate experiments shown in A; statistical analysis was non-parametric (Mann-Whitney test). [file 12915_2022_1305_MOESM3_ESM.pdf]

**A****DMSO****PMA**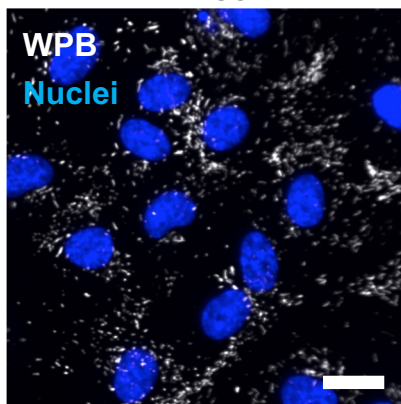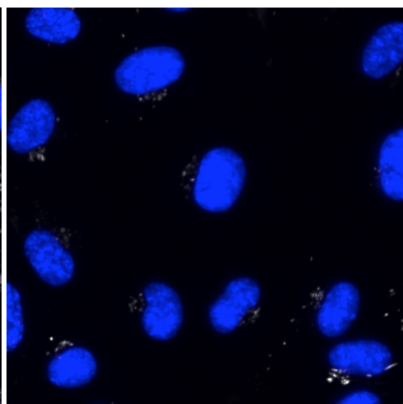**B**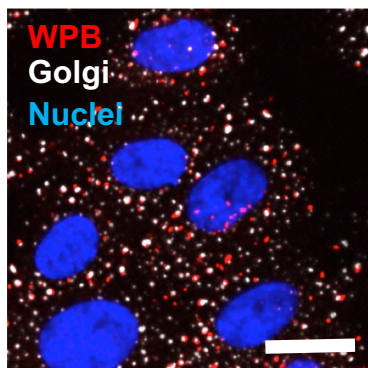**C**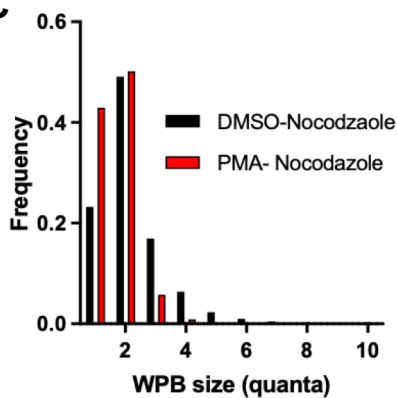**D**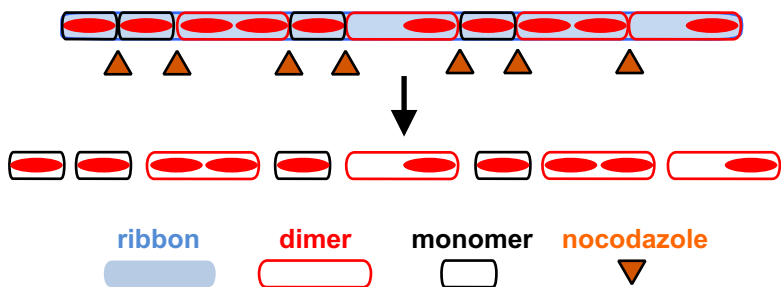

Supplement: Supplementary file 4 — Additional file 4: Fig. S4. Shifts in WPB size distribution following Golgi ribbon unlinking. A HUVECs were treated for 1 h with either DMSO or 100 ng/mL PMA and processed for immunofluorescence. PMA almost completely depletes the cellular pool of WPBs. Scale bar, 25 μm. B Representative micrograph of HUVECs pre-treated with PMA as in A to clear WPBs and then chased in nocodazole for 24 h; scale bar: 25 μm. C Size distribution of WPBs 24 h after nocodazole treatment, following pre-treatment with either DMSO (control) or PMA to deplete the organelles. The resulting populations of organelles following 24 h nocodazole treatment are newly-made WPBs plus those left after basal exocytosis, in the case DMSO; almost completely newly-made WPBs, in the case of PMA (see panel A). D Visualization of a hypothetical arrangement of the Golgi ribbon where stable mini-stacks dimers are independent of microtubules. [file 12915_2022_1305_MOESM4_ESM.pdf]
