# Supplementary material for: Structure modeling hints at a granular organization of the Golgi ribbon
Source: BMC Biol. 2022 May 13;20:111. doi: 10.1186/s12915-022-01305-3 (PMC9102599; doi:10.1186/s12915-022-01305-3)
Supplement: Supplementary file 5 — Additional file 5: Fig. S5. Modeling of mini-stack linking to form the Golgi ribbon. The possible mini-stacks associations up to pentamers are depicted (see text). [file 12915_2022_1305_MOESM5_ESM.pdf]

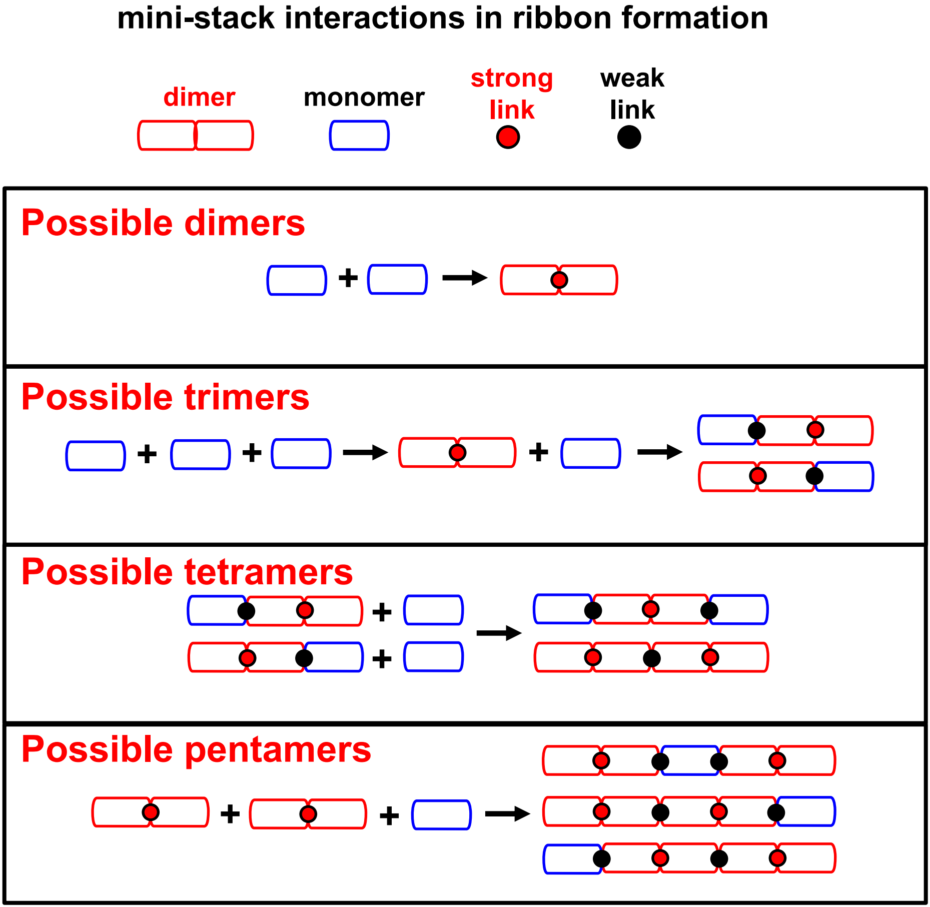
mini-stack interactions in ribbon formation
dimer
monomer
strong link
weak link
Possible dimers
Possible trimers
Possible tetramers
Possible pentamers
