# Supplementary material for: Structure modeling hints at a granular organization of the Golgi ribbon
Source: BMC Biol. 2022 May 13;20:111. doi: 10.1186/s12915-022-01305-3 (PMC9102599; doi:10.1186/s12915-022-01305-3)
Supplement: Supplementary file 6 — Additional file 6. A Reaction scheme for mini-stack linking and unlinking. B Equation system 4. [file 12915_2022_1305_MOESM6_ESM.docx]

Reaction scheme for the mini-stack binding

**A**

$$l_{1}+l_{1}\underset{\to}{r}l_{2}$$

$$l_{1}+l_{2}\underset{\to}{r}l_{3}$$

$$l_{2}+l_{2}\underset{\to}{r}l_{4}$$

$$l_{1}+l_{3}\underset{\to}{r/2}l_{4}$$

$$l_{1}+l_{3}\underset{\to}{r/2}{l'}_{4}$$

$$l_{1}+l_{4}\underset{\to}{r}l_{5}$$

$$l_{1}+{l'}_{4}\underset{\to}{r}l_{5}$$

$$l_{2}+l_{3}\underset{\to}{r/2}l_{5}$$

$$l_{2}+l_{3}\underset{\to}{r/2}{l'}_{5}$$

$$l_{2}\underset{\to}{d_{2}}l_{1}+l_{1}$$

$$l_{3}\underset{\to}{{d_{1}+d}_{2}}l_{1}+l_{2}$$

$$l_{4}\underset{\to}{d_{1}}l_{2}+l_{2}$$

$$l_{4}\underset{\to}{2d_{2}}l_{1}+l_{3}$$

$${l'}_{4}\underset{\to}{d_{2}}l_{2}+l_{2}$$

$${l'}_{4}\underset{\to}{2d_{1}}l_{1}+l_{3}$$

$$l_{5}\underset{\to}{d_{1}}l_{1}+l_{4}$$

$$l_{5}\underset{\to}{d_{2}}l_{1}+{l'}_{4}$$

$$l_{5}\underset{\to}{d_{1}+d_{2}}l_{2}+l_{3}$$

$${l'}_{5}\underset{\to}{2d_{2}}l_{1}+l_{4}$$

$${l'}_{5}\underset{\to}{{2d}_{1}}l_{2}+l_{3}$$

**B**

Equation system **4**

$$\frac{dl_{1}}{dt}=-{rl}_{1}\left( l_{1}+l_{2}{+l}_{3}+l_{4}+{l'}_{4} \right)+{2d}_{2}l_{2}+\left( d_{1}+d_{2} \right)l_{3}+2d_{2}l_{4}+2d_{1}{l^{'}}_{4}+\left( d_{1}+d_{2} \right)l_{5}+{{2d_{2}l}^{'}}_{5}$$

$$\frac{dl_{2}}{dt}=rl_{1}^{2}/2-{rl}_{2}(l_{1}+l_{2}{+l}_{3})-d_{2}l_{2}+\left( d_{1}+d_{2} \right)l_{3}+{2d}_{1}l_{4}+2d_{2}{l^{'}}_{4}+\left( d_{1}+d_{2} \right)l_{5}+{{2d_{1}l}^{'}}_{5}$$

$$\frac{dl_{3}}{dt}=rl_{1}l_{2}-rl_{3}\left( l_{1}+l_{2} \right)-\left( d_{1}+d_{2} \right)l_{3}+2d_{2}l_{4}+2d_{1}{l^{'}}_{4}+2d_{1}{l^{'}}_{5}+\left( d_{1}+d_{2} \right)l_{5}$$

$$\frac{dl_{4}}{dt}=\frac{rl_{1}l_{3}}{2}+\frac{rl_{2}^{2}}{2}-rl_{4}l_{1}-\left( d_{1}+{2d}_{2} \right)l_{4}+d_{1}l_{5}+2d_{2}{l'}_{5}$$

$$\frac{d{l'}_{4}}{dt}=rl_{1}l_{3}/2-r{l'}_{4}l_{1}-\left( {2d}_{1}+d_{2} \right)l_{4}^{'}+d_{2}l_{5}$$

$$\frac{dl_{5}}{dt}=rl_{1}{(l}_{4}+{l'}_{4})+rl_{2}l_{3}/2-2\left( d_{1}+d_{2} \right)l_{5}$$

$$\frac{d{l'}_{5}}{dt}=\frac{rl_{2}l_{3}}{2}-2\left( d_{1}+d_{2} \right){l^{'}}_{5}.$$
